# Refeeding syndrome and psychopharmacological interventions in children and adolescents with Anorexia Nervosa: a focus on olanzapine-related modifications of electrolyte balance

**DOI:** 10.1007/s00431-024-05430-9

**Published:** 2024-02-12

**Authors:** Jacopo Pruccoli, Elena Barbieri, Caterina Visconti, Beatrice Pranzetti, Ilaria Pettenuzzo, Filomena Moscano, Elisabetta Malaspina, Marastella Marino, Beatrice Valeriani, Antonia Parmeggiani

**Affiliations:** 1 IRCCS Istituto delle Scienze Neurologiche di Bologna, Centro Regionale per i Disturbi della Nutrizione e dell’Alimentazione in Età Evolutiva, U.O. Neuropsichiatria dell’Età Pediatrica Bologna, Bologna, Italy; 2 Dipartimento di Scienze Mediche e Chirurgiche (DIMEC), Università di Bologna, Via Massarenti 9, 40138 Bologna, Italy; 3 Centre for Chronic Intestinal Failure - Clinical Nutrition and Metabolism Unit, IRCCS Azienda Ospedaliero-Universitaria di Bologna, Bologna, Italy

**Keywords:** Anorexia Nervosa, Refeeding Syndrome, Hypophosphatemia, Hypokalemia, Hypomagnesemia, Psychopharmacological treatment, Olanzapine

## Abstract

This study aims to investigate the potential correlation between the use of olanzapine, a psychopharmacological intervention commonly prescribed in Anorexia Nervosa treatment, and the occurrence of Refeeding Syndrome. Despite the acknowledged nutritional and biochemical impacts of olanzapine, the literature lacks information regarding its specific association with Refeeding Syndrome onset in individuals with Anorexia Nervosa. This is a naturalistic, retrospective, observational study, reporting the occurrence of Refeeding Syndrome in children and adolescents with Anorexia Nervosa, treated or untreated with olanzapine. Dosages and serum levels of olanzapine were assessed for potential associations with the occurrence of Refeeding Syndrome and specific variations in Refeeding Syndrome–related electrolytes. Overall, 113 patients were enrolled, including 46 (41%) who developed a Refeeding Syndrome. Mild (87%), moderate (6.5%), and severe (6.5%) Refeeding Syndrome was described, at a current average intake of 1378 ± 289 kcal/day (39 ± 7.7 kcal/kg/die), frequently associated with nasogastric tube (39%) or parenteral (2.2%) nutrition. Individuals receiving olanzapine experienced a more positive phosphorus balance than those who did not (F(1,110) = 4.835, *p* = 0.030), but no difference in the occurrence of Refeeding Syndrome was documented. The mean prescribed doses and serum concentrations of olanzapine were comparable between Refeeding Syndrome and no-Refeeding Syndrome patients.

*    Conclusion*: The present paper describes the occurrence of Refeeding Syndrome and its association with olanzapine prescriptions in children and adolescents with Anorexia Nervosa. Olanzapine was associated with a more positive phosphorus balance, but not with a different occurrence of Refeeding Syndrome. Further, longitudinal studies are required.

**Table:** 

**What is Known:** *• Refeeding Syndrome (RS) is a critical complication during refeeding in malnourished patients, marked by electrolyte (phosphorus, magnesium, potassium) imbalances.* *• Olanzapine, an atypical antipsychotic with nutritional and biochemical impacts, is used in Anorexia Nervosa (AN) treatment, however data concerning its association with RS are lacking.*	
**What is New:** *• The study observed RS in 46/113 (41%) young patients with AN.* *• Olanzapine-treated individuals showed a higher improvement in serum phosphate levels than untreated ones, although no impact on the occurrence of Refeeding Syndrome was observed.*

**What is Known:**

*• Refeeding Syndrome (RS) is a critical complication during refeeding in malnourished patients, marked by electrolyte (phosphorus, magnesium, potassium) imbalances.*

*• Olanzapine, an atypical antipsychotic with nutritional and biochemical impacts, is used in Anorexia Nervosa (AN) treatment, however data concerning its association with RS are lacking.*

**What is New:**

*• The study observed RS in 46/113 (41%) young patients with AN.*

*• Olanzapine-treated individuals showed a higher improvement in serum phosphate levels than untreated ones, although no impact on the occurrence of Refeeding Syndrome was observed.*

## Introduction

Anorexia Nervosa (AN) is a feeding and eating disorder (FED) defined by restriction of energy intake, leading to significantly low body weight relative to age, sex, developmental trajectories, and physical health [1].

In severely malnourished patients, intensive enteral or parental feeding may lead to the development of Refeeding Syndrome (RS), a condition characterized by metabolic and electrolyte imbalances that can cause cardiac arrhythmia, cardiac failure, delirium, seizures, coma, and sudden death [2–4].

Due to the lack of a universally agreed-upon definition of RS in the literature, the American Society for Parenteral and Enteral Nutrition (ASPEN), Parenteral Nutrition Safety Committee, and the Clinical Practice Committee introduced a new clinical RS definition [2]:

- A mild RS is indicated by a 10–20% decrease in serum phosphorus, potassium, and/or magnesium levels; a moderate RS by 20–30% decrease; and a severe RS by a decrease greater than 30% and/or organ dysfunction resulting from a reduction of the above electrolytes or thiamine deficiency.
- These alterations should occur within 5 days of reinitiating or substantially increasing energy intake.

Treatment protocols to address RS-specific complications for individuals with AN have recently been proposed [5].

Despite limited evidence supporting their effectiveness in treating the core symptoms of FED, the use of psychopharmacological interventions is increasing in children and adolescents with AN [6–8]. Olanzapine, an atypical antipsychotic (AAP), has been reported to reduce anxiety and cognitive rigidity and promote weight gain [6–8]. These effects are linked to improved regulation of the serotonergic and dopaminergic systems, as well as interactions with metabolic receptors, including histamine H1 receptors influencing appetite [9, 10]. These effects could impact the risk of RS by improving appetite and facilitating regular food reintroduction. Numerous studies have investigated the connection between RS and alterations in weight [11, 12] and appetite [13] in individuals with FED. Pathophysiological consequences of olanzapine treatments, moreover, should be acknowledged by clinicians. Olanzapine has been found to possess direct cardiac electrophysiological effects, since it may prolong cardiac repolarization [14]; this should be taken in significant consideration, given the documented cardiac abnormalities that may independently present in children and adolescents with AN [15]. However, research on the occurrence of RS in individuals undergoing olanzapine treatment is lacking.

This article aims to assess the occurrence of RS in children and adolescents with AN at a third-level Italian Center for FED, exploring possible associations with olanzapine treatment.

## Materials and methods

### Study design and participants

This naturalistic, retrospective observational study was conducted within the framework of an observational research that aimed to investigate the utilization of psychopharmacological treatments in a tertiary-level Italian Regional Center specialized in FED in Children and Adolescents. It received approval from the local ethical committee under the code NPI-DAPSIFA2020. The study adhered to the Strengthening the Reporting of Observational Studies in Epidemiology (STROBE) guidelines both during the planning and implementation phases [16]. This study did not receive any sponsorship or funding from external companies.

The research was carried out in July 2022 and involved a retrospective analysis of patients who had been assessed at the study Center during the period from January 1, 2016, to December 31, 2020, and had undergone at least one hospitalization due to FED at the same Center. Hospitalization, in this context, refers to either inpatient or day hospital treatment. Notably, the day-hospital treatment program for patients with FED is designed to be equally structured and intensive as inpatient treatment. The hospital’s treatment protocol involves a comprehensive, multidisciplinary approach that encompasses psychological, psychopharmacological, and nutritional interventions, conformed to established clinical international guidelines. The treatment protocol adopted in the management of the patients here included has been discussed in two previous papers published by our group, reporting the endocrinological [17] and psychopathological [18] features of the population admitted to our Center; the same population was assessed to be included in this research, with study-specific inclusion and exclusion criteria.

For patients diagnosed with AN, the Center provides various nutritional therapies, including oral dietary plans, nutritional supplements, nasogastric tube feeding (NGT), and parenteral nutrition. The oral diet emphasizes balanced meals for weight management, while nutritional supplements are offered to those who may have difficulty meeting their nutritional requirements. NGT is employed in cases of severe undernourishment or when oral feeding presents challenges. In circumstances where alternative approaches are unfeasible, parenteral nutrition is administered intravenously. These interventions are personalized to each patient’s specific needs and are the result of careful assessments and consultations with the multidisciplinary team.

The Center serves a dual role, providing psychiatric hospital care for AN and acting as the primary healthcare facility for individuals with severe underweight conditions and metabolic impairment. While the primary approach is to avoid parenteral nutrition, this intervention is reserved for exceptional cases when alternative interventions are impractical or insufficient. To provide a naturalistic depiction of RS incidence and severity, it is important to note that parenteral nutrition is considered a potential risk factor. Despite its impact on electrolyte balance, we intentionally included such cases to comprehensively assess challenges and outcomes, offering a holistic understanding of the real-world clinical scenario.

Concerning daily caloric intake, at our Centre clinicians usually started refeeding with 15–20 kcal/Kg/day in the first 24 h for patients at intermediate risk, followed by a progressive increase every 2–3 days based on plasma electrolyte levels and clinical circumstances. To reduce the risk of underfeeding and refeeding syndrome, high-risk patients consumed 5–10 kcal/kg/day of calories on the first days, increasing to a maximum of 20 kcal/kg/day in the first weeks. More rapid, AN-specific, protocols for renutrition have been published after patients enrolled in this study were hospitalized [5].

To be eligible for inclusion in this study, individuals had to meet the following criteria: (a) a diagnosis of AN based on the criteria outlined in the DSM-5; (b) the provision of informed consent; (c) a documented history of hospitalization at our Center during the relevant time frame; and (d) two blood samples, including all the three RS-involved electrolytes (phosphorus, potassium, magnesium). The exclusion criteria in this study were (a) the absence of sufficient clinical documentation, (b) a first blood sample taken more than 1 day after the start of the treatment program, and (c) a second blood sample taken more than 1 month after the first blood sample or occurring more than 5 days of reinitiating or substantially increasing energy provision.

Patients with evidence of RS were then included in the RS group according to the ASPEN criteria [2]. Individuals who did not develop RS were included in the no-RS group. The selection of the 2 groups was performed including all the patients consecutively undergoing the same hospital treatment during the selected period, to provide an unbiased and naturalistic observation. Given the naturalistic nature of the study, missing data were not replaced.

### Assessment methods

Patients underwent a thorough assessment for FED, covering psychopathological, nutritional, and biochemical screenings upon hospital admission. Collected data included demographic information, clinical factors (AN subtype, comorbidities, duration of untreated illness, use of nasogastric tube feeding, and parenteral nutrition), and anthropometric parameters (admission and discharge %BMI and BMI). The reference values for calculating %BMI (BMI/median BMI for age and gender × 100) were taken from the World Health Organization’s tables [19].

AN diagnoses and comorbidities were determined by specialists using DSM-5 criteria. Standardized laboratory tests, including blood counts and electrolyte assessments, were conducted throughout hospitalization.

Detailed data on RS were recorded, including energy intake (kcal/kg/day) and electrolyte levels, before and during RS. Electrolyte changes were measured ad a percentage between initial and subsequent samples. The laboratory methods used for blood measurements included UV photometry for phosphorus, indirect potentiometry for potassium, and colorimetric kinetic method for magnesium. Laboratory reference values were as follows: phosphorus, 2.5–4.5 mg/dl; potassium, 3.5–5.3 mmol/L; and magnesium, 1.6–2.6 mg/dl.

Variables related to psychopharmacological treatments were assessed by thoroughly reviewing the clinical documentation, which included details about the timing and duration of treatment, initial and maximum dosages, any reasons for discontinuation of treatment, and potential adverse drug reactions (ADR) that may have arisen. It is essential to note that all patients received a multidisciplinary hospital-based treatment for AN, which encompassed both individual and group psychotherapeutic interventions.

### Statistical analysis

The enrolled patients were categorized into two distinct groups based on the occurrence of RS during their respective hospitalizations. Descriptive analyses were conducted for the entire study population as well as for the two specified groups. A significance level of 0.05 was established for all statistical tests, and two-tailed tests were employed. To evaluate the normality of data distribution and the homogeneity of variance, Shapiro–Wilk’s and Levene’s tests were utilized. The two groups were subsequently compared using the Chi-Square test for categorical variables (with the Fisher exact test being employed when necessary due to small sample sizes) and the Student’s T-test for continuous variables (Mann–Whitney *U* Test was used for non-normally distributed data). The percentage of variation for the ASPEN-relevant electrolytes involved in RS (phosphorus, potassium, magnesium) was compared between patients treated and untreated with olanzapine. To account for the potential confounding effect of baseline electrolyte levels, this analysis was conducted by performing analyses of covariance (ANCOVA) corrected for baseline electrolytes (phosphorus, potassium, and magnesium, respectively). Finally, the potential admission-discharge modifications in %BMI were compared between patients treated and untreated with olanzapine, with a baseline %BMI-corrected ANCOVA.

The determination of the sample size was based on the number of individuals enrolled during the study period. Given the retrospective design of this study focusing on the naturalistic portrayal of real-world data, missing data were not imputed or replaced. This approach was employed to maintain the originality and unaltered nature of the dataset, aligning with the study’s aim to authentically reflect the clinical scenario under investigation. All the statistical analyses were carried out using JASP (Jeffrey’s Statistical Program), version 17.1 for Windows.

## Results

### Enrollment procedures

A total of 330 children and adolescents diagnosed with FED, who sought care at the Center during the designated period, were initially identified and considered for inclusion in the study. Their mean age was 16 years with a standard deviation of 3.3, and the cohort included 26 males, constituting 7.9% of the total sample.

Following the application of the inclusion criteria, 251 children and adolescents diagnosed with AN, who had accessed our Center during the specified period and had a documented history of hospitalization, were selected for inclusion in the study. Then, exclusion criteria were applied. Both patients with a first blood sample taken more than 1 day after the start of the treatment program, as well as patients with a second blood sample taken more than 1 month after the first blood sample, or more than 5 days of reinitiating or substantially increasing energy provision were excluded. Thus, 113 patients were retained in the final analyses, with 67 (59%) patients not developing an RS and 46 (41%) who developed an RS.

### Patients’ characteristics

Patients’ characteristics are summarized in Table 1. No statistically significant difference was documented between the RS group and the no-RS group for demographics and clinical variables. Particularly, the two groups were comparable for their BMI and %BMI at admission.

**Table 1:** Descriptive statistics for the two groups RS group and the no-RS group

**Variables**	***RS (n = 46)***	***no-RS (n = 67)***	**Significance**
**Demographics**			
Gender	F = 42 (91%) M = 4 (8.7%)	F = 60 (90%) M = 7 (10%)	*p* = 1.000
Age (years)	15 (IQR = 3)	15 (IQR = 3)	*p* = 0.403
Duration of untreated illness (months)	11 (IQR = 9)	9 (IQR = 11)	*p* = 0.169
**Anthropometric**			
Admission %BMI	71 (± 10)	72 (± 10)	*p* = 0.361
Discharge %BMI	79 (IQR = 11)	80 (IQR = 13)	*p* = 0.182
Admission BMI (kg/m^2^)	14 (IQR = 2)	14 (IQR = 3)	*p* = 0.689
Discharge BMI (kg/m^2^)	15 (IQR = 2)	16 (IQR = 3)	*p* = 0.356
**Clinical**			
AN diagnosis	Restrictive AN = 41 (89%) Binge-purging AN = 5 (11%) Atypical AN = 0 (0%)	Restrictive AN = 62 (93%) Binge-purging AN = 3 (4.5%) Atypical AN = 2 (3%)	*p* = 0.225
Major depressive disorder	1 (2.2%)	4 (5.9%)	*p* = 0.647
Obsessive–compulsive disorder	4 (8.7%)	6 (9.0%)	*p* = 1.000
Nasogastric tube feeding	18 (39%)	26 (39%)	*p* = 0.972
**Antipsychotics (at the moment of the 2nd blood sample)**			
Atypical antipsychotic	13 (28%)	25 (37%)	*p* = 0.317
Olanzapine	5 (11%)	14 (21%)	*p* = 0.162
Aripiprazole	4 (8.7%)	7 (10%)	*p* = 1.000
Risperidone	3 (6.5%)	3 (4.5%)	*p* = 0.686
Quetiapine	2 (4.4%)	2 (3%)	*p* = 1.000
**Antidepressants (at the moment of the 2nd blood sample)**			
SSRI	27 (59%)	39 (58%)	*p* = 0.959
Sertraline	19 (41%)	34 (51%)	*p* = 0.323
Fluoxetine	6 (13%)	4 (5.9%)	*p* = 0.312
Fluvoxamine	2 (4.4%)	1 (1.5%)	*p* = 0.566

*AN* Anorexia Nervosa, *BMI* body mass index, *%BMI* body mass index percentage, *SSRI* selective serotonin reuptake inhibitors

Psychopharmacological agents administered at the moment of the second blood sample (used to measure the occurrence of RS) are reported. No significant difference in the frequency of administration of any specific drug, including olanzapine, between the two groups was documented.

### Data concerning the occurrence of Refeeding Syndrome

Forty-six patients (41%) developed RS according to the ASPEN criteria. Among these patients, 23 (50%; 20% of the whole sample) had a decrease in serum phosphorus levels, 22 (48%; 20% of the whole sample) in serum potassium levels, and 14 (30%; 12% of the whole sample) in serum magnesium levels. Forty (87%) individuals presented mild RS, 3 (6.5%) patients developed moderate RS, and 3 (6.5%) patients presented severe RS. Electrolyte levels for the two groups at the first and second blood samples are reported in Table 2. The two groups differed for all the selected parameters, except for 2nd sample-related magnesium levels.

**Table 2:** Electrolyte variations for patients experiencing (RS) and not experiencing (no-RS) a Refeeding Syndrome

**Variables**	***RS (n = 46)***	***no-RS (n = 67)***	**Significance**
First sample electrolytes			
Phosphorus (mg/dL)	4.0 (IQR = 0.6)	3.8 (IQR = 0.6)	*p* = 0.005*
Potassium (mmol/L)	4.4 (IQR = 0.5)	4.2 (IQR = 0.4)	*p* = 0.011*
Magnesium (mg/dL)	2.3 (IQR = 0.3)	2.2 (IQR = 0.2)	*p* = 0.002*
Second sample electrolytes			
Phosphorus (mg/dL)	3.6 (IQR = 0.6)	4.2 (IQR = 0.6)	*p* < 0.001*
Potassium (mmol/L)	4.1 (IQR = 0.4)	4.3 (IQR = 0.3)	*p* < 0.001*
Magnesium (mg/dL)	2.2 (IQR = 0.3)	2.1 (IQR = 0.2)	*p* = 0.576
Percentual variations between the two blood samples			
Phosphorus (%)	− 15 (IQR = 7.5)	+ 8.3 (IQR = 15)	*p* < 0.001*
Potassium (%)	− 13 (IQR = 3.2)	0 (IQR = 14)	*p* < 0.001*
Magnesium (%)	− 13 (IQR = 4)	− 4.4% (IQR = 11)	*p* < 0.001*

*RS* Refeeding Syndrome. Statistically significant differences are marked with an asterisk and underlined

The average energy intake during RS was 1378 ± 289 kcal/day (39 ± 7.7 kcal/kg/die). This was not significantly different from what was administered to patients in the no-RS group (1357 ± 231 kcal/day; 38 ± 11 kcal/kg/die) (*p* = 0.659). Among the patients with RS, 18 (39%) were receiving supplementary enteral feeding via NGT. Among those, 16 (89%) showed mild RS and 2 (11%) severe RS. One (2.2%) individual received supplemental parenteral nutrition, with an occurrence of mild RS. Three patients among those with moderate or severe RS and 22 patients with mild RS have received electrolyte supplements.

### Refeeding Syndrome (variations in electrolyte levels) and administration of olanzapine

Potential associations between treatment with olanzapine and a variation in electrolyte levels between the first and the second blood samples were assessed. Individuals treated with olanzapine experienced significantly different evolution of serum phosphorus between the two blood samples, showing a more positive phosphorus balance (+ 11%, IQR = 14) when compared to those who did not receive olanzapine (+ 2.4%, IQR = 19) (F(1,110) = 4.835, *p* = 0.030, after correcting for baseline phosphorus levels). No significant difference was documented for potassium (*p* = 0.970) and magnesium (*p* = 0.709).

Relevantly, the mean doses of olanzapine at the moment of the second blood sample were comparable between the two groups (RS, median = 5 mg, IQR = 2.5; no-RS, median = 5 mg, IQR = 2.5; *p* = 0.972).

Serum concentrations of olanzapine were 17 ± 12 mg/dL for the RS group and 28 ± 13 mg/dL for the no-RS groups (reference laboratory values: 20–80 mg/dL). The difference between these values was not significant (*p* = 0.146).

Individuals treated with olanzapine did not experience a significantly different increase in %BMI scores at discharge compared to those untreated (*p* = 0.535).

## Discussion

In this study, we examined clinical and treatment variables among young patients with AN who developed RS during hospital treatment.

Out of our sample, 41% of patients developed RS based on ASPEN criteria (87% mild cases, 6.5% moderate, and 6.5% severe); 50% had a decrease in serum phosphorus, 48% in serum potassium, and 30% in serum magnesium levels. Our findings, showing a 20% incidence of phosphate-related RS, are in contrast with O’Connor and Nicholls’ systematic review, which reported a refeeding-related hypophosphatemia incidence of 14% in adolescents with AN [20]. Cioffi and colleagues recently reported an RS incidence ranging from 0 to 62%, with no significant change when applying the ASPEN criteria [21]. When comparing our results to previous studies, it is relevant to consider our patient population, which received a higher prevalence of psychopharmacological interventions, potentially indicating a higher level of general psychopathology and disordered eating behavior related to FED.

Unlike previous research showing a correlation between lower BMI and RS risk [22, 23], we found no significant BMI difference between the RS and the no-RS group. To assess this, clinicians should consider that our study population consisted of hospitalized patients with substantial nutritional deficiency (mean BMI 14 kg/m2, mean %BMI 72.1%).

While there was no significant difference in RS occurrence between those treated and untreated with olanzapine, our study suggested a potential association between olanzapine use and electrolyte balance. In fact, the use of olanzapine may have contributed to a more controlled reintroduction of nutrients, reducing metabolic and electrolyte imbalances, as indicated by improvements in serum phosphate levels during refeeding (higher serum phosphorus balance between blood samples 1 and 2 in olanzapine treated-patient). Despite this hypothesis, notably, individuals treated with olanzapine did not show a significant difference in admission-discharge %BMI, when compared to those untreated, thus suggesting that the effect of olanzapine is not exerted on a mere facilitation of eating and reintroduction of calories. Previous studies suggest that patients with AN treated with olanzapine may experience decreased mealtime agitation, weight-related anxieties [24], eating disorder-related behaviors, and physical hyperactivity [25]. A good tolerability profile may be reached in this population using low doses of olanzapine [25, 26]. Nonetheless, a more positive phosphorus balance does not guarantee a better clinical prognosis. Further research is needed to understand the mechanisms and establish the long-term efficacy and safety of olanzapine in this context.

We must acknowledge the limitations of this study: first of all, the retrospective and monocentric design, potentially limiting the generalizability of our findings. Secondly, more recent treatment protocols have been developed in this field in the last years. Finally, between blood samples 1 and 2, unaccounted clinical variables might have influenced electrolyte balance, potentially confounding intergroup comparisons. Nevertheless, our study offers significant strengths. We employed the internationally accepted ASPEN criteria to identify RS, enhancing the reliability and validity of our results. Additionally, our research represents the first exploration of the relationship between RS and psychopharmacological therapies, paving the way for further investigation. Furthermore, our inclusion of a relatively large sample size significantly augments the limited existing data on RS. This comprehensive overview guides future research in this field.

## Conclusion

This study, conducted at a specialized Italian Center for FED, investigated the occurrence of RS in children and adolescents with AN and explored the association with olanzapine treatment.

ASPEN criteria were used to define RS. Unlike previous research, we found no significant BMI difference between the RS and the no-RS group. Nevertheless, our findings suggest that the use of olanzapine might have played a role in facilitating a more controlled reintroduction of nutrients, thereby reducing metabolic and electrolyte imbalances. Further research is needed to fully understand the role of olanzapine and other psychopharmacological therapies in reducing RS risk; however, this study highlights the potential impact of such approaches in managing RS in this vulnerable population.

## Data Availability

The data sets used and analyzed during the current study are available from the corresponding author on reasonable request.

## Abbreviations

AAP: Atypical antipsychotic
ADR: Adverse drug reaction
AN: Anorexia Nervosa
ASPEN: American Society for Parenteral and Enteral Nutrition
BMI: Body mass index
DSM-5: Diagnostic and Statistical Manual of Mental Disorders, Fifth Edition
FED: Feeding and eating disorder
IQR: Interquartile range
NGT: Nasogastric tube feeding
RS: Refeeding Syndrome
STROBE: Strengthening the Reporting of Observational Studies in Epidemiology
